# Changing the mindset for precision medicine: from incentivized biobanking models to genomic data

**DOI:** 10.1017/S0016672319000077

**Published:** 2019-10-31

**Authors:** Daniel Tigard

**Affiliations:** RWTH Aachen University, Human-Technology Center, Applied Ethics with Focus on Ethics of Technology, Theaterplatz 14, Aachen 52062, Germany

**Keywords:** biobanking, data ethics, precision medicine, research ethics

## Abstract

The emerging paradigm in contemporary healthcare, *precision medicine*, is widely seen as a revolutionary approach to both clinical treatment and overall health promotion. Precision models are making use of the most up-to-date technological advancements – such as genomics and ‘big data’ processing – in an effort to tailor healthcare to each individual. Yet the list of hurdles to successful implementation of precision medicine is no secret. Among the challenges, it was recently suggested in this journal that we must change the ‘mindset’ of patients, practitioners and the wider public (McGonigle, 2016). And while precision medicine indeed demands a significant shift, we must not understate the extent of the overhaul required. In particular, I argue, against McGonigle's suggestion, that the ethical challenges regarding participant contributions cannot be tackled by relying upon existing models of incentivized blood banking or organ donation. Instead, the success of precision medicine requires a wholescale change in mindset.

## 1. Introduction

In 2015, President Obama's Precision Medicine Initiative propelled forward a new approach to healthcare research and clinical treatment. Moving away from a ‘one-size-fits-all’ model, the announcement and US$215 million investment endorsed ‘an innovative approach to disease prevention and treatment that takes into account individual differences in people's genes, environments, and lifestyles’ (White House, 2015). Since then, a variety of companies and medical schools, private and public alike, have broadened the notion of *precision medicine* and rolled out platforms supporting *precision health* that directly reflect the 2015 initiative. The prevailing thought is that technological advancements – such as genomics and ‘big data’ processing – will enable us to tailor healthcare to each individual, for example, by identifying genetic biomarkers of diseases.

As we might expect, the list of hurdles to successful implementation of precision medicine has been growing, from technical and legal concerns over data security to ethical issues regarding how to amass and operationalize an open database consisting of patients’ electronic medical records, lifestyle information and genetic data. Among these challenges, it was recently suggested in this journal that we must change the ‘mindset’ of patients, healthcare practitioners and the wider public (McGonigle, 2016). It is argued that precision medicine depends, perhaps paradoxically, on collective participation, and accordingly that we will need to encourage unprecedented collaborative efforts and extensive ‘citizen science’ (Prainsack, 2014; McGonigle, 2016). And while precision medicine indeed demands a significant shift in mindset, we must not understate the extent of the overhaul required. In particular, I argue, against Ian McGonigle's suggestion, that the ethical challenges regarding participant contributions cannot be tackled by relying upon existing models of incentivized blood banking or organ donation. Instead, the success of precision medicine requires a wholescale change in mindset.

## 2. Ethical tensions

In its narrower guise, precision *medicine* aims to provide treatment that will be most effective and bear the fewest undesirable side effects, while hopefully also improving economic outcomes for individual patients (Chen *et al.*, 2016). Broadening the use of such a ‘revolutionary’ approach, precision *health* platforms tend to emphasize the importance of healthy lifestyles, disease prevention and risk awareness (Akdis & Ballas, 2016). Yet, here it may seem that the precision model is not an entirely newfound approach. In their recent work on precision in critical care, Shihab Sugeir and Stephen Naylor note that Hippocrates, well over two millennia ago, ‘believed that disease was a product of environmental forces, diet and lifestyle habits, and that treatment should focus on patient care (prevention) and prognosis (prediction)’ (Sugeir & Naylor, 2018). As Sugeir and Naylor submit, personalized patient care has eroded into a protocol-based practice. What we may be seeing then, with the rise of precision models, is a return to a very classic form of medicine, albeit with the use of up-to-date tools.

Granted, the precision approach will make use of previously unheard-of social and behavioural information, and it stands to usher in a reclassification of diseases based upon one's molecular profile (Mirnezami *et al.*, 2012). Determining how to properly harness the trove of highly sensitive information – how to ensure privacy and security of the data – is naturally among the key hurdles (Mega *et al.*, 2014; Jameson & Longo, 2015). Many commentators simply acknowledge the most apparent concern, namely that we will need to balance public safety with maximized productivity (e.g., Ashley, 2015). Others have suggested that the public health benefits are unclear, that gene-based drugs will cost *more* due to targeting smaller populations and accordingly that we should temper the narrative of ‘transformative change’ (Joyner & Paneth, 2015). Nonetheless, as things stand, the US National Institutes of Health (2019a) All of Us research program and its awardees have been encouraging patients to contribute medical records and biological samples in an effort to amass a database of at least one million individuals.

As of August 2019, the number of participants in the National Institutes of Health research programme has reached approximately 245,000 (National Institutes of Health, 2019b). By contrast, the now well-known private direct-to-consumer genetic testing company 23andMe reported the genotyping of their one millionth customer in June 2015, months before the US Precision Medicine Initiative was officially launched (Wojcicki, 2015; Stoeklé *et al.*, 2016). According to one report, 23andMe is ‘amassing one of the largest genetic bio/databanks in the world’ as its ‘back-end business model’ (Spector-Bagdady, 2016). Unsurprisingly, the company has faced setbacks but also approvals on certain genetic testing from the US Food and Drug Administration (Check Hayden, 2017). And although private ownership of sensitive personal data might be less alarming under the pretext of governmental regulation, concerns are being raised over vulnerabilities from foreign access and commercial interests (e.g., Berger & Schneck, 2019).

Along with the concerns over data privacy, security, ownership and use, questions of how to justify non-therapeutic research on human subjects appear equally challenging. Here, we see one of the most fundamental tensions in research ethics: that is, determining the extent to which using a selection of individuals for the purpose of benefiting others can be morally permissible. We might suppose there simply is no problem where researchers have obtained the most robust sort of informed consent available. However, as numerous authors have made clear, the traditional standards of obtaining informed consent do not map onto secondary, future uses of subjects’ data. Indeed, consent procedures are often highly questionable – for example, due to obscurities or omissions in terms and conditions documents (Stoeklé *et al.*, 2016). For these reasons, newly proposed models of informed consent attempt to articulate a continuous, open-ended agreement to studies for which subjects’ data were not intended and which were likely unknown at the time of initial data-gathering (e.g., Hansson, 2006; Steinsbekk, 2013; Ploug & Holm, 2016). Undoubtedly, precision medicine, with its reliance upon future uses of enormous quantities of data, aptly raises such concerns for informed consent. But even if we determine an appropriate recourse for traditional consent models, a procedurally prior enquiry remains. How, if at all, can we conscript subjects in the first place? To what extent should we incentivize the voluntary donations of sensitive data?

## 3. Historical precedents?

In a recent contribution to this journal, McGonigle notes that the success of precision medicine depends upon the voluntary participation of healthy individuals, which, in turn, calls for changing the mindset of patients, healthcare providers and the wider public. Specifically, we will ‘need to embrace the “idea” that genetic information is an important part of medical treatment’ (McGonigle, 2016). In order to promote this idea, we must foster collaborations – for example, between scientific and clinical communities – and increase public engagement. Yet, as McGonigle readily points out, the ethical conundrums are substantial, including questions of genetic data ownership, risks of sharing family data and uncertainties over future withdrawals of one's data.

As a way to ‘tackle’ the ethical concerns, McGonigle suggests making comparisons with historical precedents. The first analogy on offer is a system of voluntary blood donation in Israel, wherein donors ‘receive a government identity card assigning them priority to receive future emergency blood donations’ (McGonigle, 2016). Similarly, on Israel's organ donation plan, those who sign the ‘Adi’ card agree to ‘donate their organs after death’ to help save patients awaiting transplants, and in return, donors and their relatives are granted priority for transplant wait-lists (McGonigle, 2016). Undoubtedly, there is an intuitive appeal to such schemas, reinforced with reflection upon desert-based justice and the allocation of scare resources to the contributors and their family members. According to some research, this type of incentivized biobanking is rather effective in promoting an increase in voluntary contributions (Stoler *et al.*, 2016).

However, as a precedent for operationalizing precision medicine, the blood and organ donation models understate the extensive change in mindset required. In short, incentivized blood and organ donation systems, such as those seen in Israel, are unlike the data donations needed for precision medicine. At least three important differences can be promptly clarified. First, human blood and vital organs are properly considered scarce and non-renewable resources. Patients’ medical records and genetic information, on the other hand, may be presently unavailable – particularly for legitimate use in large-scale shared databases – but they are surely not scarce in the same sense or for the same reasons. Unlike blood and organs, personal data are very easily duplicated, transmitted and shared with anyone, anywhere. Once data are *consumed*, they do not diminish in value (Hand, 2018). For this reason, data can be used again and again, well into the future, bringing a much greater risk of harm to the ‘data donor’. Second, then, it seems that the further into the future one's data are used, the less likely one knows exactly how they are being used, for what purpose and so on. In this way, the risks of data donation – regardless of novel forms of consent that may be obtained – are simply incommensurable to the risks associated with the donation of blood or organs.

Third, and perhaps most glaringly, are the incongruous incentives attached to biobanking models that offer distinct and tangible rewards. As McGonigle showed, on Israel's systems, donors clearly benefit by being prioritized for potential reception of blood or vital organs. Donors benefit their families by allowing relatives to be prioritized. These sorts of immediate rewards simply cannot be translated to incentivizing data donation, nor should they be. It would be far less clear how to be prioritized as a beneficiary of precision medicine. McGonigle notes that data donors can benefit in terms of ‘access to personal health assessment, and … by helping the wider community become healthier’ (McGonigle, 2016). However, healthcare is not a commodity with which we should reward those who submit sensitive personal data, and those inclined to support public health surely have clearer opportunities – for example, through immunization efforts or drug and nutrition awareness (Joyner & Paneth, 2015). Further, even if such incentive schemas are translated to precision medicine, incentivizing data donation appears morally wrong in ways that Israel's biobanking systems are not. Personal data and genetic information are, of course, highly sensitive. In the wrong hands, one's data can disrupt insurance premiums, employment and much more (Stiles & Appelbaum, 2019). With the donation of blood or donating organs after death, one no longer *needs* these resources. By contrast, unless we agree to donate data only upon death, we remain in need of these delicate resources even after donation.

It might be thought that incentivizing the contribution of health records and genetic data is morally permissible where subjects can identify with the goals of research or, at least, are not directly coaxed into contributing by the researchers themselves (e.g., Jonas, 1969; Macklin, 1981). In a recent report on the Genes for Good (2019) programme, for example, it is emphasized that altruism is among the primary incentives and that recruitment occurred ‘organically, with participants publicizing the study through their own networks’ – namely, Facebook (Brieger *et al.*, 2019). Of course, questions should still be raised concerning the protection of human subjects and the use of social media as a tool for research and provision of healthcare (e.g., Pedersen & Kurz, 2016; Omaggio *et al.*, 2018). It is with good reason that we see recent institutional efforts to implement professional guidelines on the use of social media, such as the American Medical Association's policy (Kind, 2015). Still, the technical, legal and ethical hurdles of big data in healthcare are far from resolved. Given the extraordinary technological advancements and the extent of the risk involved, we must take care not to mislead potential data donors into thinking the challenges of precision medicine are anything like what we have seen before.

## 4. Conclusion

McGonigle states that the benefits of precision medicine databases ‘may still be unknown, and perhaps at this point inestimable’ (McGonigle, 2016). This acknowledgement alone is informative of precision medicine's unprecedented challenges. To be sure, I have not argued that we should not voluntarily participate in the creation of databases with which we might better treat individual patients and more effectively promote the overall health of populations. The potentially widespread benefits may indeed be substantial. What I have suggested is that the challenge of amassing the large-scale databases necessary for the success of precision medicine cannot be met by looking to existing models of blood and organ donation wherein contributors are incentivized with distinct personal benefits. Clearly, the success of precision medicine requires a change in mindset on behalf of patients, practitioners and the general public. With this change, we must move away from historical precedents, away from the search for personal benefits and towards brand new ways of collectively improving individual treatment and overall health.
